# Metabolomic Evidence for Bee-Driven Detoxification, Nutritional Remodeling, and Geographic Homogenization in Rapeseed Floral Products

**DOI:** 10.3390/metabo16060403

**Published:** 2026-06-10

**Authors:** Wei-Min Gao, Chun-Mei Xiong, Jing-Juan Li, Lu Lu

**Affiliations:** 1 School of Nursing, Kunming Medical University, Kunming 650500, China; gaoweimin@kmmu.edu.cn; 2 Yunnan Provincial Key Laboratory of Pharmacology of Natural Products, School of Pharmaceutical Sciences, College of Modern Biomedical Industry of Yunnan Province, Kunming Medical University, Kunming 650500, China; 20240205@kmmu.edu.cn

**Keywords:** untargeted metabolomics, rapeseed pollen, bee pollen, honey, bee processing

## Abstract

**Highlights:**

**What are the main findings?**

- Untargeted metabolomics profiled 1308 metabolites across rapeseed raw pollen, pollen-containing anther, bee pollen and honey; bee bioprocessing remodels pollen metabolome via biotransformation, eliminating toxic alkaloids/pesticides and accumulating beneficial phospholipids and carbohydrates.
- Geographical metabolite discrepancies largely disappear after bee transformation: differential metabolites decrease from 49–53 in raw pollen to only eight in bee pollen, with distinct functional pathway enrichment among four matrices.

**What are the implications of the main findings?**

- Bee processing realizes natural detoxification and nutritional optimization of plant pollen, scientifically validating the superior safety and nutrient balance of commercially available bee pollen.
- This metabolic evidence differentiates application positioning: raw pollen for characteristic bioactive extraction, bee pollen as balanced nutritional supplement, honey as energy-supplying functional food.

**Abstract:**

**Background:** Rapeseed (*Brassica napus* L.)-derived pollen, pollen-containing anther, bee pollen, and honey are natural health products with both nutritional and functional value. Bee processing plays a key role in the dynamic transformation process of bee product formation. Existing research has mostly focused on static analyses of single product types and has not systematically elucidated the hierarchical differences in metabolites from plant pollen to bee products, the remodeling mechanism during bee processing, or the impact of geographical origin and agricultural practices on product metabolic profiles. These research gaps limit the precise development and quality control of rapeseed-derived bee products. **Methods:** This study employed UPLC-MS/MS-based untargeted metabolomics to analyze differential metabolites among rapeseed pollen, pollen-containing anther, bee pollen, and honey and elucidated functional differences via KEGG pathway enrichment analysis. **Results:** A total of 1308 metabolites were identified, primarily consisting of active components such as flavonoids and terpenoids. Origin-related differences caused by agricultural practices and companion plants were significant in raw pollen but were markedly attenuated by bee processing. Bee pollen showed increased levels of phospholipids and sugars along with reduced toxic substances, forming a safer and more balanced nutritional profile, making it suitable as a nutritional supplement. Honey was highly enriched in monosaccharides and disaccharides, combining flavor with functionality, and is suitable for the development of energy foods. **Conclusions:** Bee processing actively reshapes the pollen metabolome, attenuates geographical origin differences in raw materials, and enhances the safety and nutritional balance of bee products.

## 1. Introduction

In recent years, pollen and its bee products have gained increasing application in the functional food sector due to their richness in various bioactive substances [1]. Numerous studies have provided evidence for the application value of bee products. For instance, researchers have identified a total of 89 compounds in 20 types of bee pollen. Among these, 31 phenolamides and their isomers were detected in 17 bee pollen varieties, including those from apricot, pear, and rose, whereas 25 flavonoid glycosides were identified in 18 bee pollen types, such as corn, watermelon, and lotus [2]. Another study identified 259 compounds in the samples of bee pollen based on High-Resolution Mass Spectrometry and found translation. *Camellia* bee pollen has been reported to contain abundant polyphenolic glycosides and amino acid derivatives, while rapeseed bee pollen was found to contain a high content of ferulic acid, and corn pollen was rich in polyphenols such as naringenin [3]. A total of 43 features were associated with increased antioxidant activity and about 30 compounds, including organic acids, free amino acids, and Amadori compounds, that early Maillard reaction products were identified [4]. Liu et al. identified 61 different metabolites in six types of honey. In *Bauhinia championii* honey, 7-O-methylchrysin was identified as a characteristic marker and exhibited excellent DPPH and ABTS radical scavenging abilities due to its richness in flavonoids and mineral elements such as potassium and sodium [5]. Collectively, these studies have established a solid foundation for understanding the phytochemical basis of bee products.

However, the composition and bioactivity of bee pollen are not a simple summation or concentration of those from plant pollen; honey bees play a crucial role in this dynamic transformation process. Their behaviors—encompassing collection, hydration, plasticization, maturation, and storage, which essentially drive a complex bioprocessing cascade, resulting in significant metabolic differences between pollen and bee pollen. Recent studies have indicated that pollen and bee pollen exhibit significant differences in physical structure, microbial communities, enzyme activity, and the content and forms of certain free amino acids, sugars, and vitamins [6,7]. However, several critical aspects of the formation of bee pollen remain to be elucidated. Firstly, analyzing a single product could not determine whether key metabolites with potential health-promoting properties originate from the plant or from bee modification, nor does it systematically elucidate their metabolic formation mechanisms [8,9,10]. Similarly, for beneficial or detrimental components, it remains difficult to distinguish whether such compounds originate from inherent raw-material variation or from active biotransformation during bee processing, making it unclear what the role of bee processing in quality and safety enhancement is [11,12]. Secondly, the cross-stage dynamic metabolic changes in pollen secondary metabolites during the transition from plant origin to bee products have not been elucidated, resulting in an incomplete understanding of the mechanisms underlying the formation of product nutritional functions [13,14,15]. Therefore, conducting correlative metabolomics research on a specific pollen type and its bee products, systematically comparing the metabolic profiles among different product types and their formation mechanisms, is crucial for comprehensively elucidating the metabolic pathways from “plant raw material” to “bee-processed product,” supporting comprehensive evaluation of bee product quality, safety, and functional potential. Untargeted metabolomics enables the detection of dynamic changes in pollen metabolite profiles across different processing stages, thereby providing methodological support for investigating the remodeling effect of bee processing and the evolutionary patterns in geographical variations.

Rapeseed (*Brassica napus* L.) is one of the important nectar and pollen source plants in the Brassicaceae family. The pollen, bee pollen, and honey derived from rapeseed are recognized as natural products with significant nutritional and functional value [16]. According to Grand View Research (2025) [17], the global bee pollen market size reached $833.9 million in 2023 and is projected to grow at a compound annual growth rate (CAGR) of 6.1% from 2024 to 2030. As an important category of single-source pollen, rapeseed bee pollen accounted for approximately 28% of global single-source bee pollen sales in 2023, with an estimated CAGR (2024–2030) of 7.0%, surpassing the overall market growth rate. Rapeseed-derived bee pollen is known to contain various bioactive components, including flavonoids, phenolic acids, glucosinolates and their degradation products, terpenes, vitamins, and unsaturated fatty acids, as well as a range of volatile substances such as hydrocarbons, esters, aldehydes, ketones, and heterocyclic compounds [18,19]. These diverse components constitute the material basis for the multiple physiological functions of rapeseed bee products, including antioxidant, anti-inflammatory, antimicrobial, immunomodulatory, and potential cardiovascular protective and antitumor activities. Plant-derived metabolites are important for bee products’ functionality. However, how honey bees reshape the pollen metabolic profile during collection and in-hive transformation, and thus influence its nutritional composition and safety, remains unclear. Systematic comparative metabolomic studies on rapeseed pollen and its derived bee products can provide a scientific basis for health-related evaluations of these products [1,11]. Furthermore, the metabolic composition of raw pollen is known to be significantly influenced by geographical origin, companion plants, and agricultural practices, which may lead to substantial regional differences in nutritional components and contaminants (e.g., pesticides and alkaloids). This directly affects raw material consistency and safety risks, subsequently impacting the metabolic characteristics and functionality of the bee products [20,21]. Therefore, systematic characterization of the metabolomic profiles of rapeseed pollen at different processing stages, clarification of the targeted remodeling effect induced by bee processing, and decipherment of the evolution patterns of geographical differences along the processing chain are of great significance for comprehensively evaluating the quality, safety, and functional potential of bee products.

In this study, untargeted metabolomics technology was employed to perform a systematic comparative analysis of rapeseed pollen, pollen-containing anther, bee pollen, and honey from different geographical regions for the first time. These results revealed the dual effects of bee processing on the nutritional restructuring and geographical homogenization of the pollen metabolome, as well as an adaptive shift at the metabolic pathway level from plant reproduction and defense towards nutrient storage and energy reserves. This study aimed to clarify metabolic characteristic differences between pollen and bee products, influences of agricultural practices and companion plants on the metabolic composition of bee products, the attenuation effect of bee processing on origin-related differences, and the central role of honey bees in the formation process of bee products.

## 2. Materials and Methods

### 2.1. Sample Collection

In March 2024, four rapeseed floral products (Table S1), i.e., rapeseed pollen (D-M, *incl.* LD-M, DD-M), pollen-containing anther (DY-M, *incl.* LDY-M, DDY-M), bee pollen (FD-M, *incl.* FLD-M, FDD-M), and honey (FM-M, *incl.* LDFM-M, DDFM-M), were collected from two rapeseed cultivation sites: Laoda’aozi Village (LD, 24.98970733° N, 104.28350873° E) and Dedeng Village (DD, 24.97927012° N, 104.30488756° E) in Luoping County, Qujing City, Yunnan Province. To ensure accurate capture of the biological changes induced by the core event of bee processing and to effectively control confounding factors of the environment, we designed the sampling for each site: within the same micro-ecological area (a rapeseed field of no less than 1.5 acres), two floral products, pollen and anthers (containing pollen) from rapeseed plants at the same flowering stage were first collected. To minimize contamination from surrounding floral tissues, use a dissecting needle to cut along the sepal suture line and gently separate the sepals and petals, exposing the stamens. Use watchmaker’s forceps (type #5, tip 0.05–0.1 mm) to grasp the base of each anther. Apply a slight rotating motion while pulling upward, allowing the filament to detach naturally from its base. A healthy colony of Italian honey bees (*Apis mellifera ligustica* Spin.) was placed in the center of the same plot where fresh pollen was collected. This bee species is the predominant reared species in major nectar-producing regions of China, and its products are highly representative of the market. According to the criteria established by Nagai et al. (2005) [22], during active foraging periods, pollen pellets carried on the hind legs of bees, which had been collected from local rapeseed flowers and undergone approximately 15 days of fermentation, were gathered using an entrance pollen trap, as the third. Finally, from the same colony from which the bee pollen was collected, honey produced during the current season, predominantly sourced from rapeseed nectar, was extracted. Since pollen pellets were observed to contain honey-entrained materials during the investigation, honey was included as the fourth floral product for analysis.

Five biological replicates were established for each of four rapeseed floral products, including pollen, pollen-containing anthers, bee pollen, and honey collected from both the LD and DD locations. Immediately after collection, all samples were snap-frozen in liquid nitrogen and stored at −80 °C until metabolomic analysis. Samples were then named and grouped based on their geographical origin and type for subsequent comparative analysis.

### 2.2. Sample Preparation and Metabolomics Data Acquisition

The pollen-containing anthers were analyzed without manual separation of anther tissues and the enclosed pollen grains; the entire anther structure was processed as an intact unit. After gentle thawing of each sample type at 4 °C, an aliquot of each sample was mixed with pre-chilled methanol/acetonitrile/water (2:2:1, *v*/*v*/*v*) and vortexed to achieve homogenization. The mixture was subjected to low-temperature ultrasonic extraction for 30 min and then incubated at −20 °C for 10 min to precipitate proteins. Next, the samples were centrifuged at 14,000× *g* and 4 °C for 20 min, and the resulting supernatant was collected and vacuum-dried. Before mass spectrometry (MS) analysis, the dried residues were reconstituted in 100 μL of acetonitrile/water (1:1, *v*/*v*), vortexed thoroughly, and centrifuged again at 14,000× *g* and 4 °C for 15 min. The final supernatant was collected for instrumental analyses. Metabolite extraction, identification, and quantification were performed by Shanghai Applied Protein Technology Co., Ltd, Shanghai, China.

### 2.3. Chromatography–Mass Spectrometry Analysis

#### 2.3.1. Chromatographic Conditions

Separation was performed using an Agilent 1290 Infinity II Ultra-High Performance Liquid Chromatography (UHPLC) system (Agilent Technologies Inc., Santa Clara, CA, USA) equipped with a Waters ACQUITY UPLC BEH C18 column (1.7 μm, 2.1 mm × 100 mm) (Waters Corporation, Milford, MA, USA). The column temperature was maintained at 40 °C, with a flow rate of 0.4 mL/min and an injection volume of 2 μL. Mobile phase A consisted of water containing 25 mM ammonium acetate and 0.5% formic acid, and mobile phase B consisted of pure acetonitrile. The gradient elution program was as follows: 0–0.5 min, 5% B; 0.5–10 min, linear gradient from 5% to 100% B; 10.0–12.0 min, 100% B; 12.0–12.1 min, linear gradient from 100% to 5% B; 12.1–16 min, 5% B (equilibration). All samples were stored at 4 °C in the auto-sampler throughout the analysis. To minimize instrument signal drift, samples were injected in a randomized order, and quality control (QC) samples were interspersed within the sample sequence to monitor system stability and data reliability. QC samples were analyzed alongside biological samples to assess analytical variability. Metabolites with a relative standard deviation (RSD) < 30% in QC samples were considered reproducibly measured and retained for subsequent statistical analysis.

#### 2.3.2. Q-TOF Mass Spectrometry Conditions

An AB Sciex Triple TOF 6600 mass spectrometer (AB Sciex LLC, Marlborough, MA, USA) was used to acquire both MS1 and MS2 mass spectra. Electrospray ionization (ESI) was used in both positive and negative ion modes, with the following source parameters: Ion Source Gas 1 (GS1) = 60 psi, Ion Source Gas 2 (GS2) = 60 psi, Curtain Gas (CUR) = 30 psi, source temperature = 600 °C, and Ion Spray Voltage Floating (ISVF) = ±5500 V. The MS1 scan range was set to 60–1000 *m*/*z* with an accumulation time of 0.20 s per spectrum, whereas the MS2 product ion scan range was 25–1000 *m*/*z* with an accumulation time of 0.05 s per spectrum. MS2 data were acquired using the Information-Dependent Acquisition (IDA) mode with the following parameters: isotopes within a 4 Da window were excluded, and the top 10 most intense precursor ions per cycle were selected for fragmentation. The declustering potential (DP) was set to ±60 V, and the collision energy (CE) was optimized to 35 ± 15 eV.

### 2.4. Experimental Data Quality Assessment

Raw mass spectrometry data in Wiff format were converted to mzXML format using ProteoWizard software (version 3.0.6428). For both positive and negative ion modes, peak picking, retention time alignment, peak area normalization, and calculation of collision cross-section (CCS) values were sequentially performed using MSDIAL software (version 4.6). Data quality was assessed by evaluating the overlap of total ion chromatograms (TICs) among quality control (QC) samples to monitor instrument stability and by performing unsupervised principal component analysis (PCA) on all samples to examine overall data distribution, reproducibility, and batch effects.

### 2.5. Metabolite Identification

The data extracted by MSDAIL were used for metabolite identification against an in-house database provided by Sinobase Metabolomics (Sinobase Plant Secondary Metabolite Standard Database). Metabolite structures were identified by comparing the molecular mass errors (within <10 ppm), secondary fragmentation spectra, retention time (RT), and other information of metabolites in biological samples with those in the database. The confidence level was assigned as either Level 1 or Level 2 according to the MSI (Metabolomics Standards Initiative) guidelines.

### 2.6. Statistical Analyses

To decrease the risk of reducing variable size and losing potential differential metabolites, only metabolites with no more than 50% missing values within any sample group were retained. Subsequently, all metabolite expression levels were normalized using z-score transformation, making the data suitable for multivariate modeling. Hierarchical clustering heatmaps were generated based on Euclidean distance matrices to visualize the relative metabolite abundance across samples. Unsupervised principal component analysis (PCA) and supervised orthogonal partial least squares discriminant analysis (OPLS-DA) were performed on the normalized data for dimensionality reduction and group discrimination. To further characterize the temporal dynamics of metabolites across different stages (Y-M, FD-M, and M-M), this study performed pseudo-temporal clustering analysis using the Mfuzz package (version 2.66.0). The data processing methods and multiple analyses were adapted from those previously reported [23], with slight modifications. Specifically, differential metabolites were selected based on a VIP > 1 from the OPLS-DA model, a *p*-value < 0.05 without multiple testing correction, and an absolute fold change > 1.50 or <0.67. Differential metabolites identified in both positive and negative ion modes were merged and subjected to Kyoto Encyclopedia of Genes and Genomes (KEGG) pathway enrichment analysis. Metabolic pathways with FDR-adjusted *p*-values < 0.05 (Benjamini–Hochberg) were considered significantly enriched. The significance of pathway enrichment was calculated using Fisher’s exact test to identify metabolic and signaling pathways that were significantly perturbed across the sample groups.

## 3. Results

### 3.1. Abundant Shared Metabolites Across Different Product Types

Total ion current (TIC) analysis of quality control samples (Figure S1) indicated that the response intensities and retention times of each chromatographic peak were highly consistent, with minimal variation caused by instrumental error. Correlation analysis (Figure S2) revealed correlation coefficients > 0.9 among QC samples, confirming analytical repeatability.

**Figure 1:**
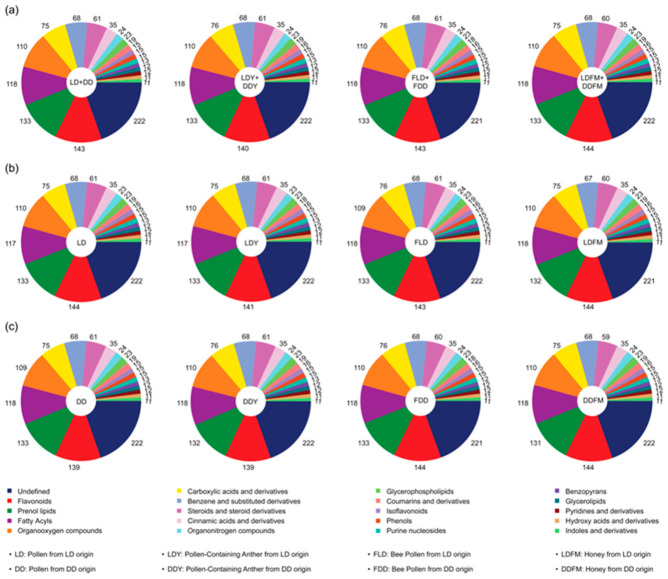
Classification and quantitative composition of the top 20 metabolite categories across sample types and origins. Due to the limited number of reference compounds in the in-house Sinobase Plant Secondary Metabolite Standard Database (provided by Sinobase Metabolomics). Metabolites not confidently identified against this database were annotated as “undefined”. (**a**) metabolite classification and quantity in four product types (pollen, pollen-containing anther, bee pollen, and honey); (**b**) metabolite classification and quantity in four product types from the LD origin; (**c**) metabolite classification and quantity in four product types from the DD origin.

A total of 1308 metabolites were identified in both positive and negative ion modes (Table S2), among which 163 metabolites were assigned to confidence level 1 and the remaining to level 2. Specifically, 1303 metabolites were identified in both pollen and pollen-containing anther samples, 1302 metabolites in bee pollen samples, and 1305 metabolites in honey samples. The relative proportional distribution of metabolite subclasses was consistent across the four product types. However, slight differences were observed in the number of metabolites in each class. Figure 1a displays the top 20 metabolite subclasses by relative abundance, along with their quantitative composition, across the four product types. Notably, minor quantitative differences were observed among sample types, including flavonoids (140 in pollen-containing anther, 143 in both pollen and bee pollen, and 144 in honey), carboxylic acids and their derivatives (76 in both pollen-containing anther and bee pollen, 75 in both pollen and honey), steroids and their derivatives (61 in pollen-containing anther, pollen, and bee pollen, 60 in honey), and coumarins and their derivatives (19 in pollen-containing anther, pollen, and honey, 18 in bee pollen). Additionally, Table S2 shows that even when the number of differential metabolites between different sample types is relatively small, the relative abundance of each metabolite varies considerably across sample types. For example, the relative abundance of batyl alcohol was more than a thousand times higher in bee pollen samples than in pollen samples.

### 3.2. Statistical and KEGG Analyses Among Four Product Types

**Figure 2:**
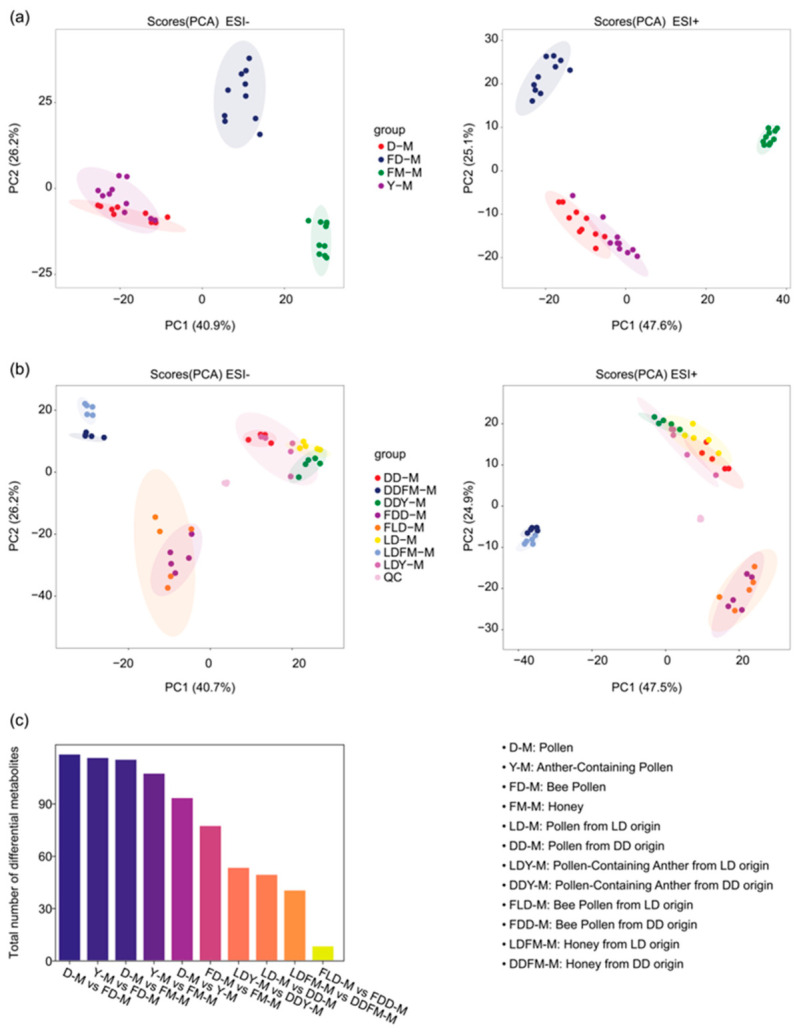
PCA results and differential metabolite statistics. (**a**) PCA score plots of four product types (pollen, pollen-containing anther, bee pollen, and honey) in positive and negative ion modes; (**b**) PCA of samples from two origins (LD and DD), with QC as quality control. (**c**) Bar chart of differential metabolite counts in each comparison group.

PCA results (Figure 2a) showed that, under both positive and negative ion modes, all samples exhibited clear clustering by product type (i.e., floral product type: pollen, anther, bee pollen, and honey). Significant separation was observed between honey, bee pollen samples, and the other product types, whereas the degree of separation between pollen and pollen-containing anther samples was relatively low. The number of significantly differential metabolites (Figure 2c, Tables S3 and S4) between bee pollen and honey (FD-M vs. FM-M, 77) was the smallest, while the numbers between pollen and bee pollen (D-M vs. FD-M, 118) and between pollen-containing anther and bee pollen (Y-M vs. FD-M, 116) were higher.

**Figure 3:**
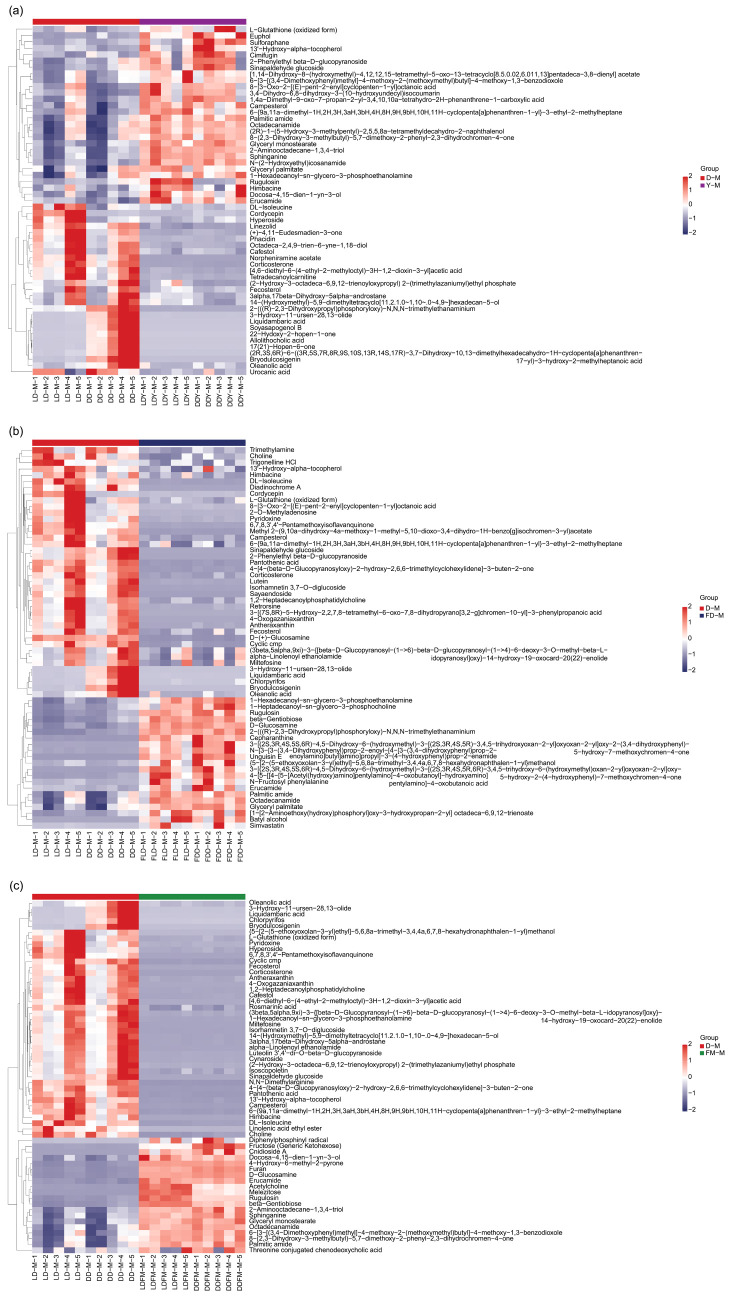
Cluster analysis of significantly differential metabolites (VIP > 1, *p* value < 0.05) identified by pairwise comparisons among four product types (pollen, pollen-containing anther, bee pollen, and honey) in positive ion mode. (**a**) D-M vs. Y-M; (**b**) D-M vs. FD-M; (**c**) D-M vs. FM-M.

Cluster analysis (Figure 3 and Figures S3–S5) revealed that, compared to pollen, the upregulated metabolites in pollen-containing anther were mainly concentrated in phenylpropanoids (e.g., 2-Phenylethyl beta-D-glucopyranoside), fatty acyls (e.g., Erucamide), organic acids (e.g., Urocanic acid), and terpenoids (e.g., Euphol), with particularly rich glycosylation modifications of flavonoid compounds (e.g., isorhamnetin-3,4’-diglucoside). In contrast, pollen samples exhibited enrichment in the synthesis and modification of terpenoids, particularly triterpenoids, such as ursolic acid, oleanolic acid, and 8-hydroxyoctadecatrienoic acid. From pure pollen to bee pollen, the lipid components in pollen, primarily free fatty acids, are replaced in bee pollen by glycerolipids, batyl alcohol, and other glyceryl ether lipids, while the content of disaccharides such as gentiobiose is significantly upregulated. Furthermore, bee pollen showed a greater variety of flavonoid core structures (e.g., kaempferol, quercetin, luteolin) and more complex glycosylation patterns in phenylpropanoid metabolism. Additionally, bee pollen exhibited a specific enrichment of sugar alcohols and amino sugars in carbohydrate metabolism and a significant downregulation of the organophosphorus pesticide chlorpyrifos, which was relatively abundant in pollen samples. Honey was characterized by the upregulation of monosaccharides and disaccharides such as lactose, glucose, and fructose, along with functional oligosaccharides including raffinose and stachyose.

**Figure 4:**
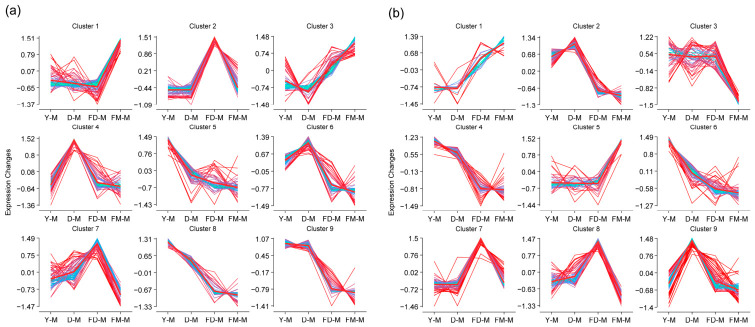
Mfuzz clustering of metabolites in negative (**a**) and positive ion modes (**b**). The *x*-axis indicates the sample types: Y-M (pollen-containing anther), D-M (pollen), FD-M (bee pollen), and FM-M (honey). The *y*-axis represents the normalized metabolite abundance (or expression level). The thick red line in each subcluster illustrates the overall expression trend of that cluster.

Under both positive and negative ion modes, Mfuzz clustering analysis identified nine distinct expression trend clusters with significant differences (Figure 4, Table S5). Among them, clusters 2 and 7 in negative ion mode (Figure 4a), as well as clusters 1, 7, and 8 in positive ion mode (Figure 4b), exhibited the highest expression levels at the bee pollen (FD-M) stage. Moreover, their overall expression levels showed a continuously increasing trend during the process of bee pollen formation (Y-M → FD-M). In addition, clusters 5, 8, and 9 in Figure 4a, along with clusters 4 and 6 in Figure 4b, displayed a continuously decreasing trend in overall expression levels throughout bee pollen formation.

**Figure 5:**
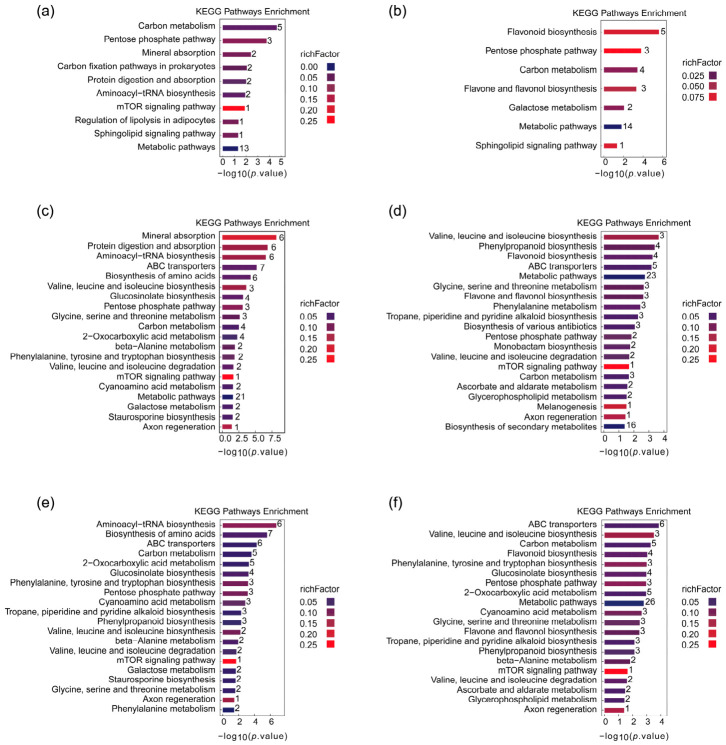
KEGG pathway enrichment bar charts for significantly differential metabolites (VIP > 1, FDR-adjusted *p*-value < 0.05) from pairwise comparisons of pollen-containing anther, pollen, bee pollen, and honey (the top 20 pathways are displayed when more than 20 are enriched). In the bar plot, the vertical axis represents each KEGG metabolic pathway, and the horizontal axis represents the *p*-value of the enrichment analysis (expressed as the negative common logarithm, i.e., −log_10_ (lg) *p*-value). (**a**) D-M vs. Y-M; (**b**) FD-M vs. FM-M; (**c**) D-M vs. FM-M; (**d**) Y-M vs. FD-M; (**e**) Y-M vs. FM-M; (**f**) D-M vs. FD-M.

KEGG pathway enrichment analysis (Figure 5) revealed that the pollen-containing anther stage was enriched in phenylpropanoid biosynthesis and the mTOR signaling pathway, reflecting the developmental and defense functions of anther tissue. The pollen stage was enriched in amino acid biosynthesis and flavonoid metabolism pathways, highlighting its role as a plant reproductive unit. The bee pollen stage was enriched in ABC transporters and glycerophospholipid metabolism pathways, indicating nutrient transport and membrane structure remodeling associated with bee processing. The honey stage was enriched in energy metabolism pathways, such as the pentose phosphate pathway and carbon metabolism, confirming its function as an energy reserve.

### 3.3. Analyses Among Different Product Types of Different Geographical Origins

#### 3.3.1. Identification of Metabolites in Different Product Types

The ranking of metabolite subclasses by quantity was consistent across sample types from the two locations, with slight differences in metabolite counts. In pollen-containing anther, 1300 metabolites were identified from both locations, but the number of flavonoids differed (141 in LDY-M and 139 in DDY-M). For pollen samples, 1303 (LD-M) and 1299 (DD-M) metabolites were identified, with flavonoid counts of 144 (LD-M) and 139 (DD-M), respectively. For bee pollen, 1303 (FLD-M) and 1302 (FDD-M) metabolites were identified, with flavonoid counts of 143 (FLD-M) and 144 (FDD-M), respectively. For honey, 1301 (LDFM-M) and 1302 (DDFM-M) metabolites were identified, with flavonoid counts of 144 in both. Figure 1b, c present the total metabolite counts in each sample type, as well as the top 20 metabolite subclasses ranked by quantity proportional abundance and their respective counts.

#### 3.3.2. Statistical and KEGG Analyses Among Four Product Types of Different Origins

PCA results (Figure 2b) showed varying degrees of separation between samples of different types from LD and DD. Specifically, bee pollen exhibited the smallest inter-location separation (FLD-M and FDD-M clustered most closely together), whereas pollen, anther, and honey samples showed clearer separation between the two geographical origins. Thus, origin-related differences varied among the same product types, with bee pollen showing the smallest such differences. The number of significantly different metabolites (Figure 2c, Tables S3 and S4) was 53 between pollen-containing anther samples from the two locations, 49 between pollen samples, 40 between honey samples, and only 8 between bee pollen samples.

**Figure 6:**
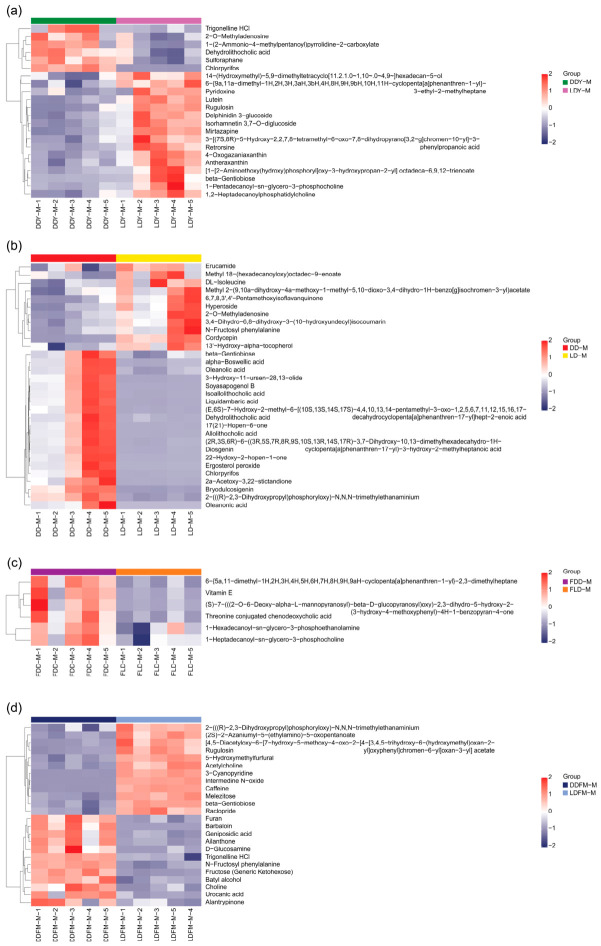
Hierarchical clustering analysis of differential metabolites (VIP > 1, *p* value < 0.05) between the same product types from LD and DD origins (positive ion mode). (**a**) LDY-M vs. DDY-M; (**b**) LD-M vs. DD-M; (**c**) FLD-M vs. FDD-M; (**d**) LDFM-M vs. DDFM-M.

Cluster analysis (Figure 6 and Figure S6) revealed that in pollen and pollen-containing anther that had not undergone bee processing, origin-related differences were extremely pronounced, with differential metabolites mainly concentrated in terpenoids, flavonoids, and contaminants. In samples from the DD location, flavanones (e.g., eriodictyol), flavonol glycosides (e.g., isorhamnetin-3-O-glucoside and rhamnetin-3-sophoroside), sulforaphane, and the organophosphorus pesticide chlorpyrifos were significantly upregulated. In contrast, samples from the LD location were primarily characterized by significant enrichment of isoflavonoids, such as eupatin and glycitein, as well as pyrrolizidine alkaloids (alkaloid class) and rugulosin (mycotoxin). Bee pollen samples from the two locations exhibited only minor differences in a few individual metabolites. Analysis of differential metabolites between honey samples from the two locations revealed that simple cyclic molecules and basic metabolites, such as furan, fructose, D-glucosamine, aloin, geniposidic acid, ailanthone, and trigonelline hydrochloride, were significantly upregulated in honey from the DD location. In contrast, upregulated metabolites in honey from the LD location were mainly highly glycosylated secondary metabolites, including structurally complex flavonoid glycosides, melezitose, and *β*-gentiobiose, as well as polyketide/anthraquinone derivatives with complex fused ring systems (rugulosin), pyrrolizidine alkaloid N-oxides, and various nitrogen-containing heterocyclic alkaloids.

**Figure 7:**
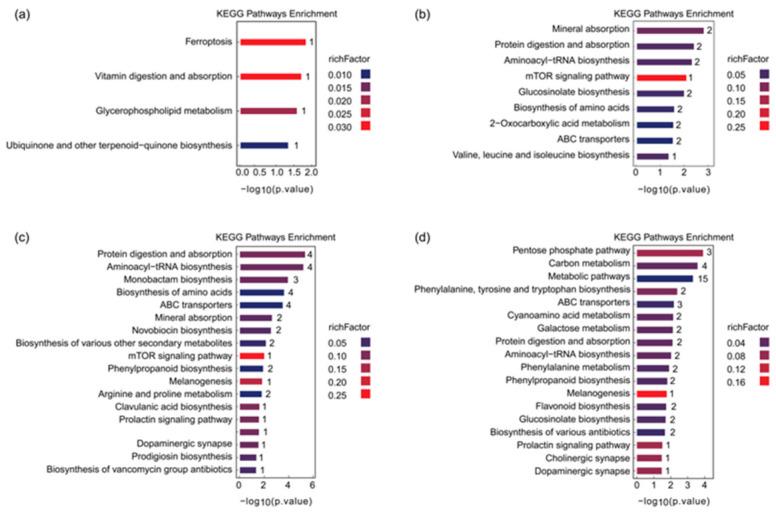
KEGG pathway enrichment bar charts for differential metabolites (VIP > 1, FDR-adjusted *p*-value < 0.05) between the same product types from LD and DD origins (the top 20 pathways are displayed when more than 20 are enriched): (**a**) FLD-M vs. FDD-M; (**b**) LD-M vs. DD-M; (**c**) LDY-M vs. DDY-M; (**d**) LDFM-M vs. DDFM-M.

KEGG pathway enrichment analysis (Figure 7) showed that the comparison of LDY-M vs. DDY-M was enriched in pathways such as phenylpropanoid biosynthesis, melanogenesis, and arginine and proline metabolism, indicating significant differences in plant defense metabolism and specific amino acid metabolism between pollen-containing anther from the two locations. LD-M vs. DD-M showed enrichment in mineral absorption, protein digestion and absorption, and aminoacyl-tRNA biosynthesis pathways, suggesting differences in basal nutrient absorption, assimilation, and anabolic metabolism. FLD-M vs. FDD-M was enriched in ferroptosis, vitamin digestion and absorption, and glycerophospholipid metabolism pathways, indicating that differences in bee pollen from the two locations were concentrated in cellular stress, vitamin utilization, and membrane lipid metabolism. LDFM-M vs. DDFM-M showed enrichment in the pentose phosphate pathway, carbon metabolism, and phenylalanine/tyrosine/tryptophan biosynthesis pathways, suggesting differences in energy metabolism and amino acid synthesis between honey from the two locations.

## 4. Discussion

### 4.1. Key Metabolites Determine Differences Between the Sample Types

A total of 1308 metabolites were identified in this study, among which flavonoids, terpenoids, and fatty acyls accounted for the highest proportions. Among these, flavonoid and phenolic amide levels are positively associated with the antioxidant activity of bee pollen [24] and are important components for bee pollen to exert its beneficial health effects, including antioxidant, anti-inflammatory, immunomodulatory, liver and cardiovascular protection, and endocrine regulation [25].

Although the four rapeseed floral products showed only minor quantitative differences in the number of metabolites (Figure 1), their major difference resides in metabolite abundance (concentrations), a finding consistently supported by many previous studies. That is to say, previous studies indicate that the metabolite profiles of homologous pollen and bee products are highly similar, differing mainly in concentrations rather than compound types. Bayram et al. (2021) [26] employed targeted metabolomics to identify 18 phenolic compounds in both bee pollen and bee feed derived from the same plant sources. Of these, 16 compounds were detected in both product types, but there were still significant differences in their quantitative results. Tomás-Barberán et al. (1989) [27] analyzed the pollen of the almond plant by using 2D PC and by HPLC coupled with a photodiode array detector. They discovered that it had the same flavonoid profile as the pollen of the almond bee pollen. The four types of products in this study are from the same plant species, hence they share a large number of metabolites, whereas significant differences were observed in the relative contents of certain metabolites across product types. Specifically, differences in lipid composition between bee pollen and pollen (Figure 3 and Figures S3–S5) reflect the nutrient reserve characteristics of bee pollen. Honey is characterized by a marked enrichment of monosaccharides and disaccharides, while retaining some flavonoids. This creates a metabolic profile that combines energy supply with functional activity. Such a profile is consistent with the phenomenon of “synergistic coexistence of sugars and antioxidant active compounds” discovered in buckwheat honey [4]. Previous studies have found that polyphenolic glycosides and amino acid derivatives are the main bioactive components in *Camellia* bee pollen. In contrast, rapeseed bee pollen exhibits a higher abundance of terpenoid lipids, particularly triterpenoids and phytosterols. This difference further confirms that the source plant species is a key factor in determining the fundamental characteristics of the pollen and bee product metabolome [9,19]. However, it is worth emphasizing that in complex biological systems, phenotypic differences are often determined by a few key biomarkers. If some compounds are detected exclusively in certain bee products or are significantly more abundant or profile-defining, they could be considered as markers of these products [28]. This indicates that the presence or absence of one or a few characteristic metabolites, along with their abundance levels, can serve as important reference indicators for assisting in the assessment of the biological characteristics of the sample.

The processes of honey bee collection, saliva mixing, and in-hive transformation may actively and directionally remodel the pollen metabolic profile. The fact that specific metabolite clusters showed sustained upward or downward trends during the entire process of bee pollen formation (Figure 4) may support this inference. The lipid compositional changes observed during this process are likely attributed to catalytic enzymes, including amylase and esterase, present in bee saliva [29]. Akiki et al. (2014) pointed out that insects often enzymatically convert dietary lipids into energy storage forms when processing plant materials [30]. The present study provides direct metabolomic evidence supporting this mechanism during bee pollen formation. The process by which honey bees collect nectar to produce honey serves as a typical example of this enzymatic transformation mechanism. Nectar oligosaccharides are primarily composed of sucrose. Through the action of enzymes like invertase and sucrase in honey bee saliva, sucrose is directionally hydrolyzed into monosaccharides such as glucose and fructose. This process is also accompanied by the specific enrichment of functional oligosaccharides such as raffinose and stachyose. As a result, honey exhibits an energy metabolism characterized by a predominance of monosaccharides and disaccharides. Furthermore, the abundant free amino acids (e.g., leucine, valine) in pollen are replaced in bee pollen by nitrogen storage forms such as asparagine, accompanied by increased levels of energy metabolism intermediates such as 3-hydroxypropionic acid, indicating that honey bees provide more readily utilizable nutritional forms through metabolic remodeling.

### 4.2. Attenuation Effect of Bee Processing on Origin-Related Differences

Geographical origin and companion plants significantly influence the metabolic composition of pollen and bee products [21], whereas bee processing attenuated these regional differences to some extent. Relatively high levels of the organophosphorus pesticide chlorpyrifos were detected in pollen from the DD location (Figure 6b and Figure S6b), suggesting higher pesticide application intensity in that area. In contrast, the significant expression of pyrrolizidine alkaloids and rugulosin in pollen from the LD location may originate from cross-pollination by companion plants or the accumulation of environmental pollutants. Gardana et al. (2018) also found that differences in companion plant species directly lead to regional differentiation of the pollen metabolome, consistent with the findings of the present study [20]. These findings emphasize that during the collection of raw bee product materials, attention must be paid not only to the growth environment of the primary nectar/pollen source plant but also to the composition and contamination status of companion plants.

Although the interpretation of the results from the KEGG pathway enrichment analysis might involve a certain degree of speculation, the results nonetheless provide substantial insights on further investigation. From the perspective of KEGG pathway enrichment analysis, LD-M vs. DD-M was significantly enriched in pathways such as mineral absorption and protein digestion and absorption, which may suggest differences in basal nutrient metabolism between pollen from the two locations. In contrast, LDY-M vs. DDY-M was enriched in pathways such as phenylpropanoid biosynthesis and melanogenesis, potentially reflecting specific differences in plant defense metabolism. These pathway differences seem to support the notion that the geographical environment indirectly shapes the metabolic composition of pollen and pollen-containing anther by influencing plant nutrient absorption and defense mechanisms. Notably, the inclusion of anthers did not alter the principal characteristics of origin-related differences. The metabolic difference patterns between pollen-containing anther from the two locations were highly similar to those of pure pollen, which further suggests that the influence of geographical factors on the pollen metabolome is far greater than minor tissue admixture.

Unlike pollen and pollen-containing anther, origin-related differences in bee pollen appeared to be significantly attenuated. Only minor differences observed in a few metabolites (Figure 2c and Figure 6c and Figure S6c), which may reflect the attenuating effect of bee processing. Specifically, when collecting pollen, bees add saliva and nectar to process it [31], while potentially also mixing in pollen from other plant sources [32]. This process may not only dilute components from a single origin but also consistently modify metabolites through enzymatic reactions. From the perspective of KEGG pathways, bee pollen from the two locations (FLD-M vs. FDD-M) was enriched only in pathways such as ferroptosis and vitamin digestion and absorption, forming a marked contrast with the extent of pathway differences in pollen. This may be due to the bee processing minimizing the impact of origin-related differences on metabolic functions. Corby-Harris et al. (2014) found that the microbial community in the crop is dominated by *Lactobacillus kunkeei* and Acetobacteraceae [33]. It may serve as a primary fermentative agent during the early formation of bee pollen. This finding provided a microbiological explanation for the attenuation of origin-related differences by bee processing.

However, origin-related differences in honey were not completely eliminated. Forty differential metabolites were identified between honey from the two locations (LDFM-M vs. DDFM-M), with significant enrichment in pathways such as the pentose phosphate pathway and carbon metabolism. This is closely related to the honey formation mechanism. The main components of honey are products of nectar and bee salivary enzymes, and the metabolic composition of nectar is directly influenced by the regional specificity of nectar source plants [34]. Additionally, regional differences exist in environmental factors, such as bee salivary enzyme composition, in-hive fermentation temperature, and humidity, further promoting metabolic divergence of honey [35,36,37,38]. Notably, the differential metabolites in honey from the two locations were mainly sugars and flavonoids, which may confer regional specificity in taste and functional activity. For example, the high content of complex flavonoid glycosides in honey from the LD location may confer stronger antioxidant activity and more pronounced bitterness and astringency [39,40], whereas the abundant simple sugars in honey from the DD location may provide a more desirable flavor profile.

### 4.3. Detoxification Effect of Bee Processing

Pesticide application in agricultural practices is a key factor affecting pollen metabolic safety. Chlorpyrifos, detected in this study, is a widely used organophosphorus insecticide. These residues not only affect the safety of pollen for consumption but may also pose potential hazards to bee health. Notably, chlorpyrifos, which was highly expressed in pollen from the DD location, was significantly downregulated in bee pollen, further confirming the detoxification function of bee processing. Harwood & Dolezal (2020) demonstrated that the cytochrome P450 enzyme system in bees can effectively degrade organophosphorus pesticides [41]. The results of this study provide direct evidence for this detoxification pathway. Furthermore, toxic substances such as pyrrolizidine alkaloids and rugulosin, which were highly expressed in pollen from the LD location, were significantly reduced in bee pollen, accompanied by a significant upregulation of prenylated and highly glycosylated flavonoid derivatives. This is consistent with the findings of Haque et al. (2025), who stated that the endogenous detoxification enzyme system is critically involved in the tolerance of honey bees to plant-derived toxins [42]. The European Union has established strict limits for pesticide residues and alkaloids in pollen and pollen products [43]. The detection of contaminants in pollen samples in this study highlights the importance of quality control of raw bee products.

## 5. Conclusions

This study, based on untargeted metabolomics analysis using UPLC-MS/MS technology, systematically revealed the metabolomic characteristics and functional differences among rapeseed pollen, pollen-containing anther, bee pollen, and honey for the first time. It also elucidated three key functions of bee processing, including metabolic remodeling, detoxification, and attenuation of geographical variation. The functional differentiation patterns of the four product types were further identified. The study found that bee processing can directionally modify the nutritional composition of pollen, enhance its safety and bioavailability, and significantly attenuate differences among raw materials from different origins, thereby stabilizing bee pollen quality. Furthermore, the four product types exhibited clear distinctions in their metabolic characteristics and functional positioning, providing a basis for differentiated product development. Future research should focus on the effects of different bee species and rearing practices on processing outcomes.

## Data Availability

The raw mass spectrometry metabolomics data and metadata have been deposited in the MetaboLights database under accession number MTBLS14321 (https://www.ebi.ac.uk/metabolights/MTBLS14321, accessed on 20 April 2026). The dataset is currently restricted and will be made publicly available on 13 April 2027. All Supplementary Tables and additional supporting data accompanying this study are provided as Supplementary Materials with the published article.

## Supplementary Materials

The following supporting information can be downloaded at https://www.mdpi.com/article/10.3390/metabo16060403/s1, Figure S1: TIC of quality control samples; Figure S2: Correlation analysis; Figure S3: Cluster analysis of differential metabolites among four product types in positive ion mode; Figure S4: Cluster analysis of differential metabolites among four product types in negative ion mode ((a) D-M vs. Y-M; (b) D-M vs. FD-M; (c) D-M vs. FM-M); Figure S5: Cluster analysis of differential metabolites among four product types in negative ion mode ((a) Y-M vs. FD-M; (b) Y-M vs. FM-M; (c) FD-M vs. FM-M); Figure S6: Hierarchical clustering of same-type samples from LD and DD in negative ion mode; Table S1: Sample information; Table S2: Metabolite identification across sample types; Table S3: Statistical summary of differential metabolites in negative ion mode; Table S4: Statistical summary of differential metabolites in positive ion mode; Table S5: Summary table of cluster membership for metabolites annotated in Mufzz analysis under positive and negative ion modes.

## Abbreviations

ABTS	2,2’-Azinobis-(3-ethylbenzthiazoline-6-sulphonate)
CAGR	Compound Annual Growth Rate
CCS	Collision Cross-Section
CE	Collision Energy
CUR	Curtain Gas
DP	Declustering Potential
DPPH	2,2-Diphenyl-1-picrylhydrazyl
ESI	Electrospray Ionization
FDR	False Discovery Rate
IDA	Information-Dependent Acquisition
KEGG	Kyoto Encyclopedia of Genes and Genomes
MS	Mass Spectrometry
OPLS-DA	Orthogonal Partial Least Squares Discriminant Analysis
PCA	Principal Component Analysis
QC	Quality Control
Q-TOF	Quadrupole Time-of-Flight
RT	Retention Time
RSD	Relative Standard Deviation
TIC	Total Ion Current
UHPLC	Ultra-High-Performance Liquid Chromatography
UPLC-MS/MS	Ultra-High-Performance Liquid Chromatography–Tandem Mass Spectrometry
VIP	Variable Importance in Projection
