# Supplementary material for: Metabolomic Evidence for Bee-Driven Detoxification, Nutritional Remodeling, and Geographic Homogenization in Rapeseed Floral Products
Source: Metabolites. 2026 Jun 10;16(6):403. doi: 10.3390/metabo16060403 (PMC13303756; doi:10.3390/metabo16060403)
Supplement: Supplementary file 1 [file metabolites-16-00403-s001.zip › supplementary file contents.pdf]

Supporting Information

Metabolomic Evidence for Bee-Driven Detoxification, Nutritional Remodeling, and Geographic Homogenization in Rapeseed Floral Products

Wei-Ming Gao[#], Chun-Mei Xiong[#], Jing-Juan Li*, and Lu Lu*

School of Pharmaceutical Sciences & Yunnan Provincial Key Laboratory of Pharmacology of Natural Products, Kunming Medical University (College of Modern Biomedical Industry of Yunnan Province), Kunming, Yunnan 650500, China

Contents

Supplementary Figures..1

Figure S1: TIC of quality control samples..1

Figure S2: Correlation analysis...1

Figure S3: Cluster analysis of differential metabolites among four product types in positive ion mode..2

Figure S4: Cluster analysis of differential metabolites among four product types in negative ion mode (D-M vs. Y-M; (b) D-M vs. FD-M; (c) D-M vs. FM-M)............................3

Figure S5: Cluster analysis of differential metabolites among four product types in negative ion mode (Y-M vs. FD-M; (b) Y-M vs. FM-M; (c) FD-M vs. FM-M).......................4

Figure S6: Hierarchical clustering of same-type samples from LD and DD in negative ion mode 5

Supplementary Tabiles ....in separate Excel file

Table S1: Sample Information.

Table S2: Metabolite identification across sample types.

Table S3: Statistical summary of differential metabolites in negative ion mode.

Table S4: Statistical summary of differential metabolites in positive ion mode.

Table S5: Summary table of cluster membership for metabolites annotated in Mufzz analysis under positive and negative ion modes.

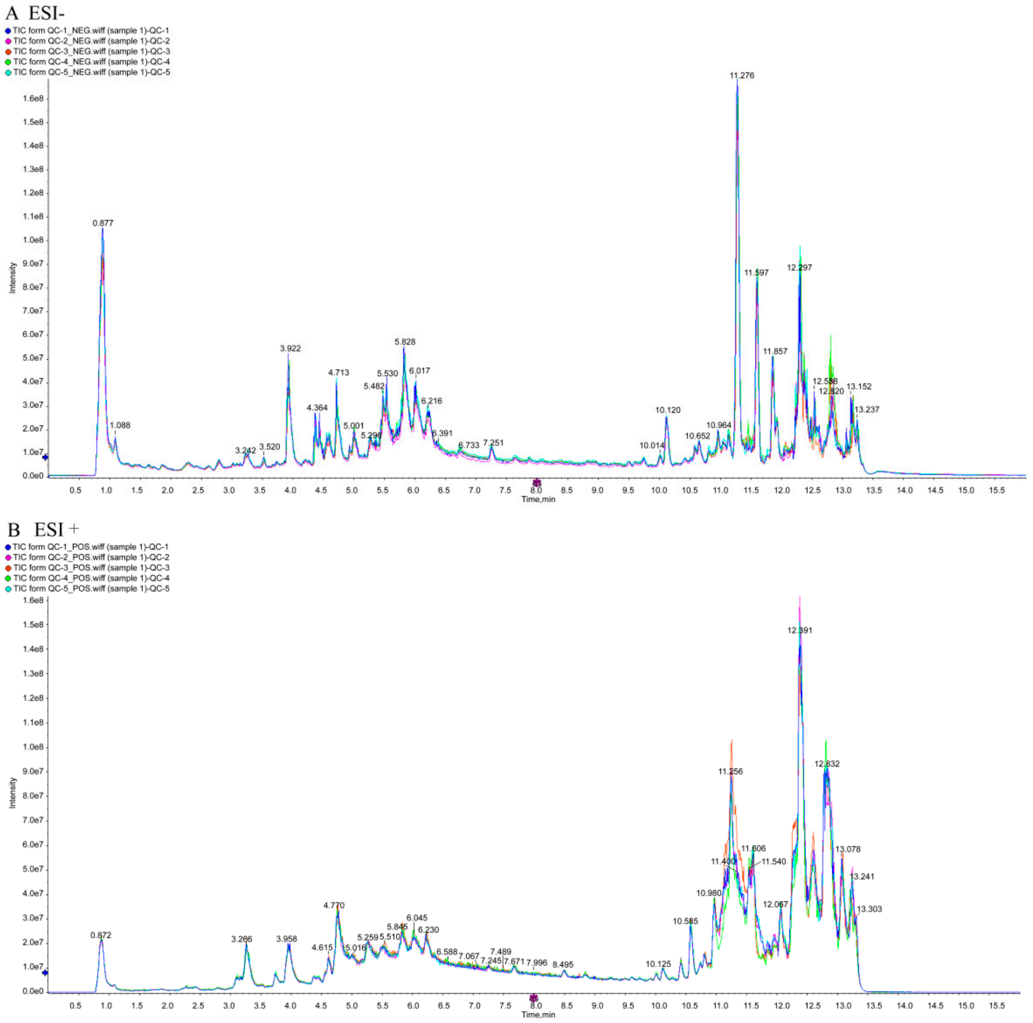

Figure S1: Total ion current chromatogram of quality control samples

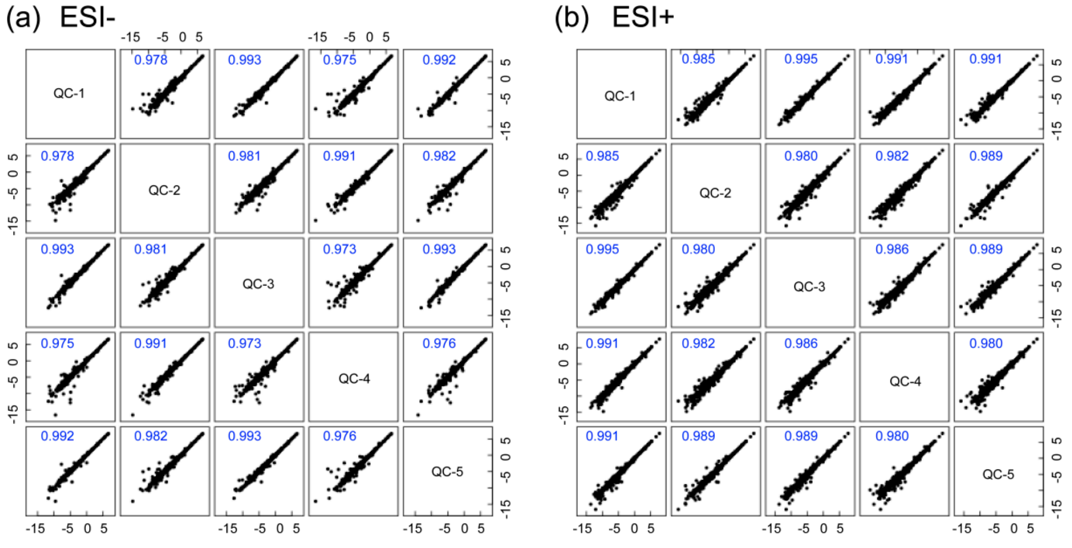

Figure S2: Correlation analysis results

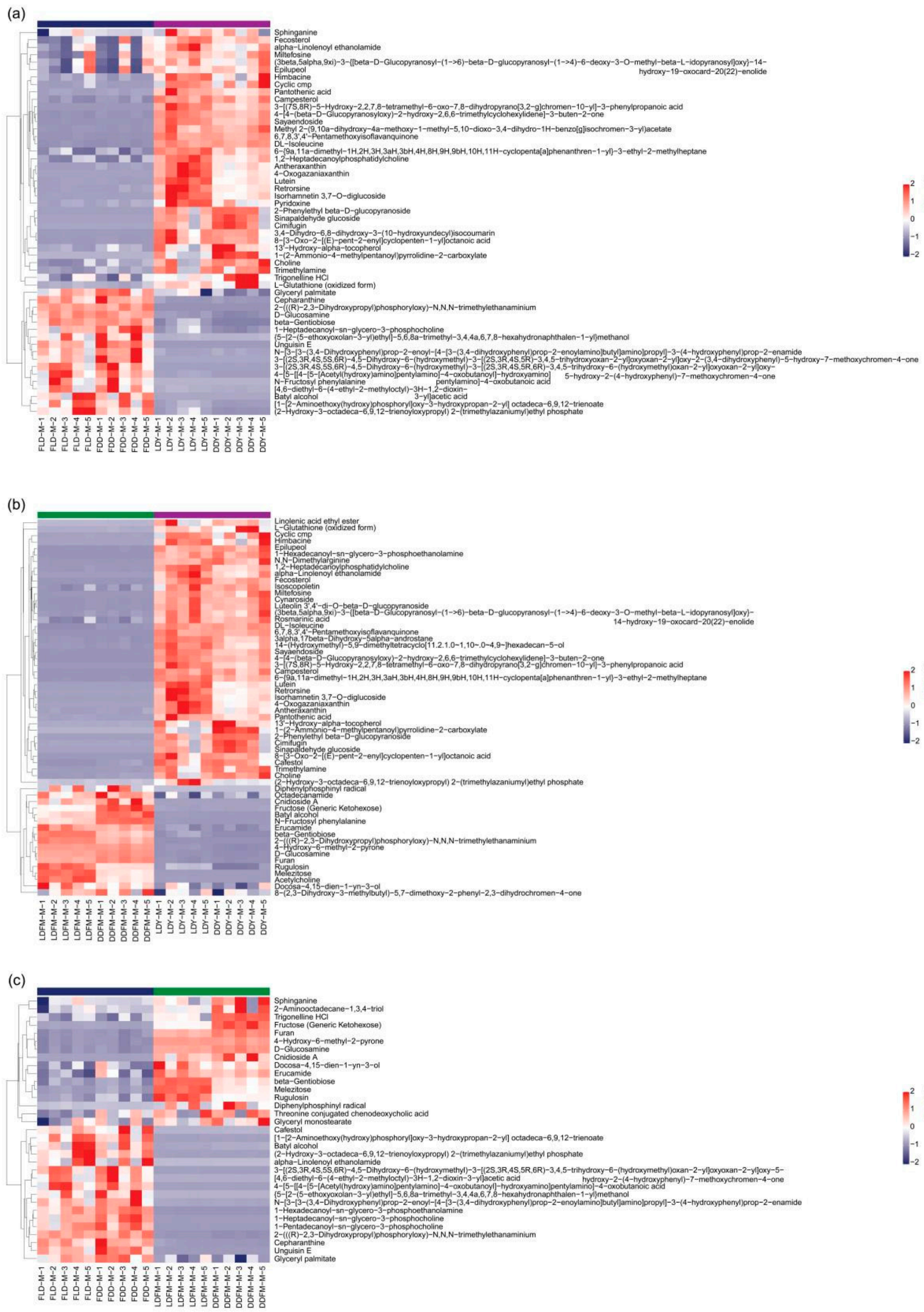

Figure S3: Cluster analysis of significantly differential metabolites identified by pairwise comparisons among four sample types (pollen-containing anther, pollen, bee pollen, and honey) in positive ion mode. (a) Y-M vs. FD-M; (b) Y-M vs. FM-M; (c) FD-M vs. FM-M.

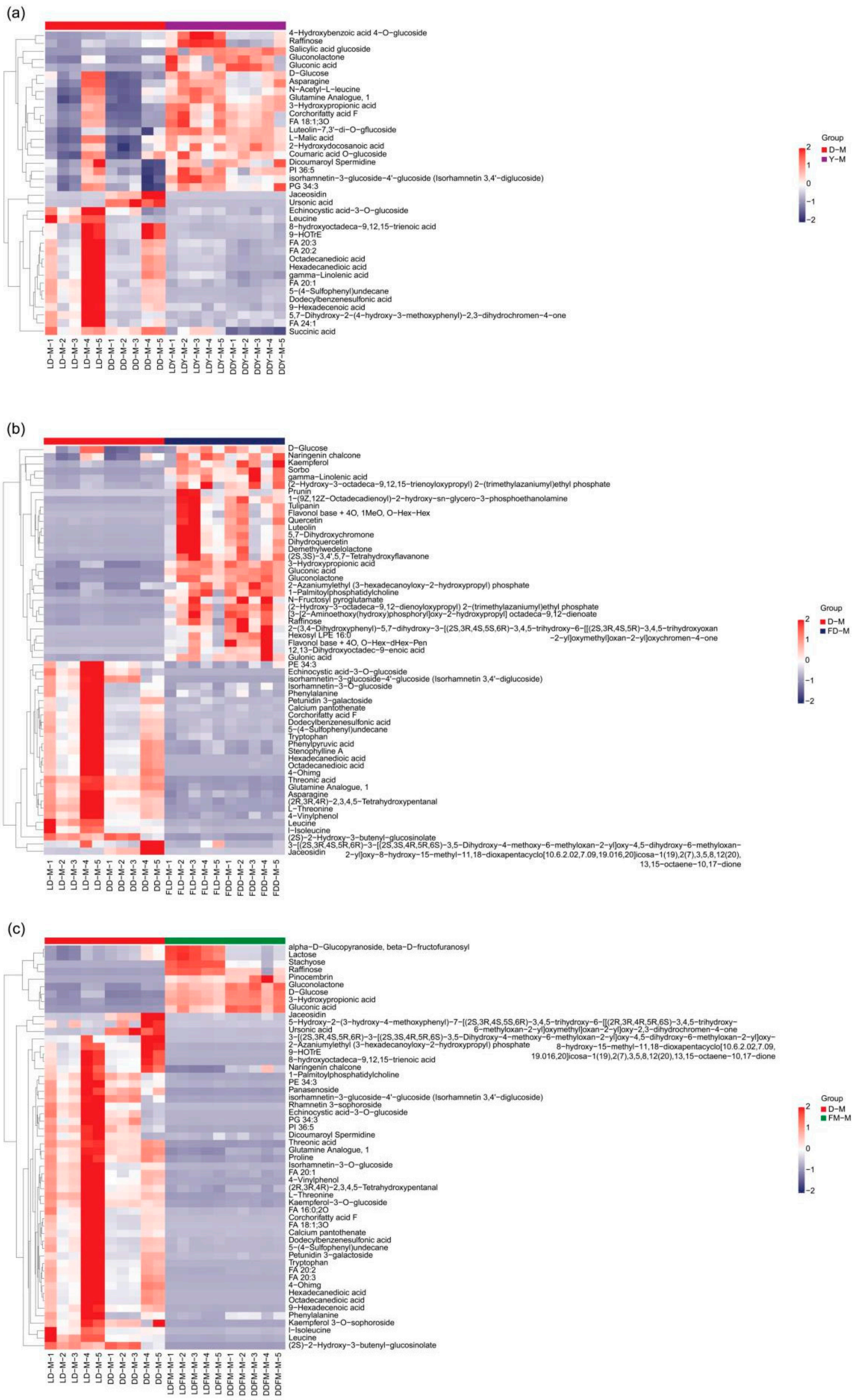

Figure S4: Cluster analysis of significantly differential metabolites identified by pairwise comparisons among four sample types (pollen-containing anther, pollen, bee pollen, and honey) in negative ion mode. (a) D-M vs. Y-M; (b) D-M vs. FD-M; (c) D-M vs. FM-M.

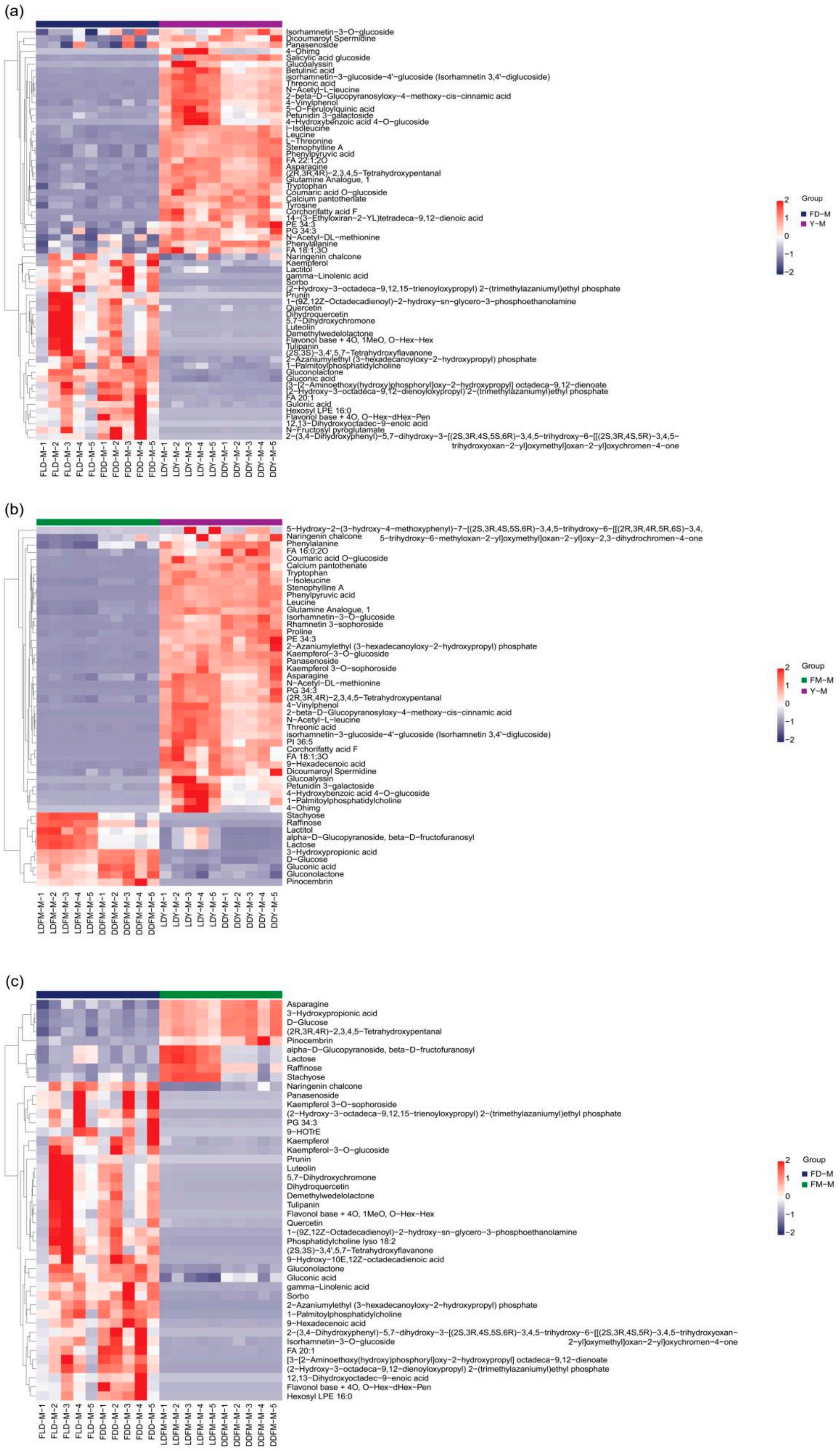

Figure S5: Cluster analysis of significantly differential metabolites identified by pairwise

comparisons among four sample types (pollen-containing anther, pollen, bee pollen, and honey) in negative ion mode. (a) Y-M vs. FD-M; (b) Y-M vs. FM-M; (c) FD-M vs. FM-M.

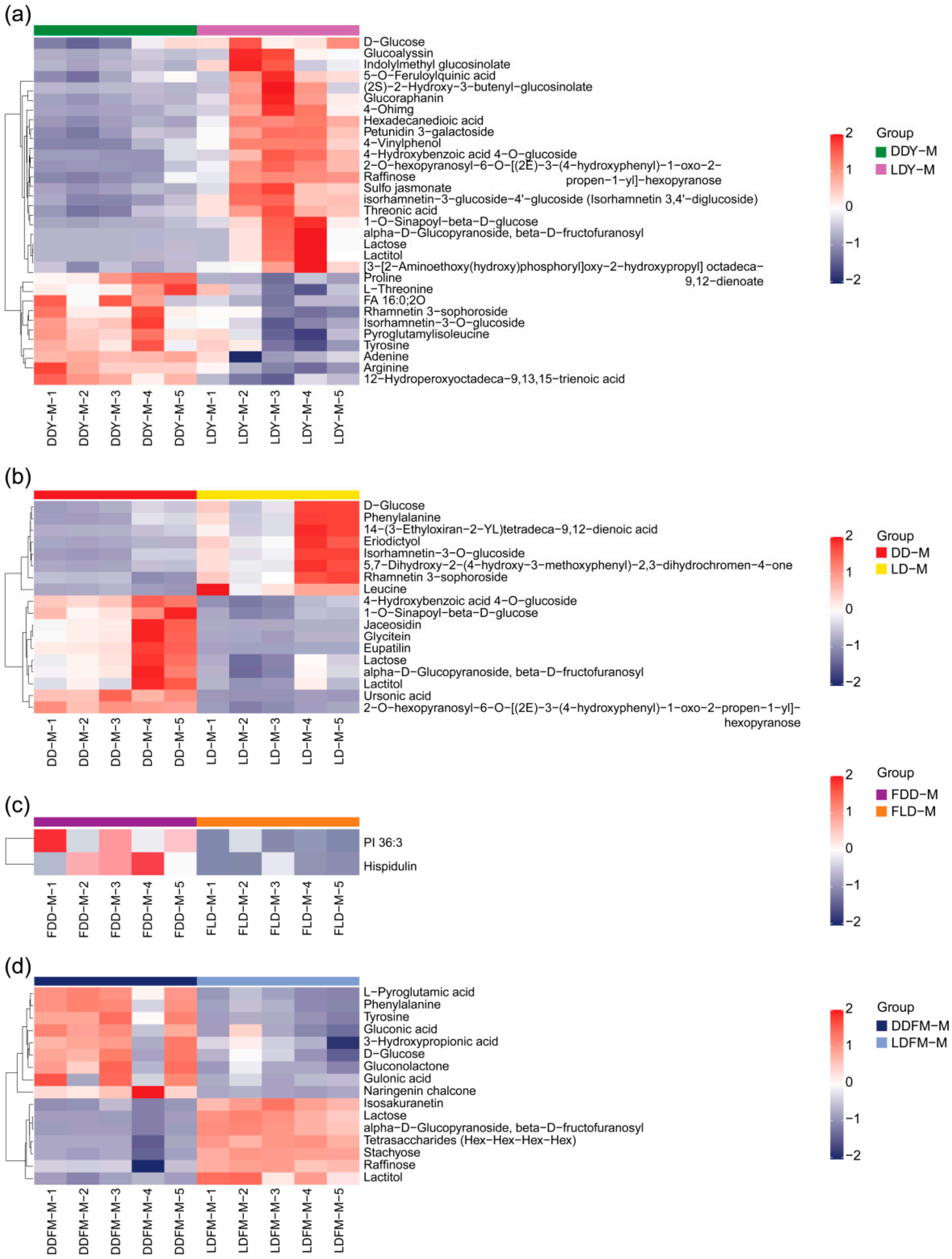

Figure S6: Hierarchical clustering analysis of differential metabolites between the same product types from LD and DD production areas in negative ion mode. (a) LDY-M vs. DDY-M; (b) LD-M vs. DD-M; (c) FLD-M vs. FDD-M; (d) LDFM-M vs. DDFM-M.
